# A spotlight on lime: a review about adverse reactions and clinical manifestations due to *Citrus aurantiifolia*

**DOI:** 10.1186/s12948-021-00152-x

**Published:** 2021-07-24

**Authors:** Clara Alessandrello, Luca Gammeri, Serena Sanfilippo, Raffaele Cordiano, Silvia Brunetto, Marco Casciaro, Sebastiano Gangemi

**Affiliations:** School and Operative Unit of Allergy and Clinical Immunology, Policlinico “G. Martino”, Department of Clinical and Experimental Medicine, University of Messina, 98125 Messina, Italy

**Keywords:** Rutaceae, *Citrus aurantiifolia*, Lime, Dermatitis, Hypersensitivity, Food allergy, Adverse reactions, Allergy, Skin

## Abstract

Lime (*Citrus aurantiifolia*) is a plant belonging to the family of Rutaceae and to the genus Citrus. The fruit is widely used in the United States, Mexico, Southeast Asia, Latin America, but is increasingly widespread all over the world. It is used as a fresh fruit, in the preparation of foods, sweets and drinks and its oils are used in the cosmetic and pharmaceutical industry. The main adverse reactions to lime seem to be represented by contact dermatitis, allergic and phototoxic type. In the context of allergic forms, several allergens have been identified in the citrus family, the main one being limonene, but no noteworthy cross-reactivity has been identified. However, a case of fruit protein contact dermatitis has been described, showing sensitization to other fruits, such as kiwi, avocado, pineapple and apple. There are several molecules responsible for phototoxic reactions and mainly belonging to the coumarin and furocoumarins families. Reactions related to ingesting the fruit or inhaling pollen from the tree appear to be rare, as there are no known cases reported in the literature. The increasing diffusion of lime in Europe must pay attention to possible adverse reactions due to contact with this fruit, which seem destined to increase in future years. Further importance must be placed on patch tests and on the possibility of using alternative extracts to classic fragrance mixes.

## Background

**Fig. 1:**
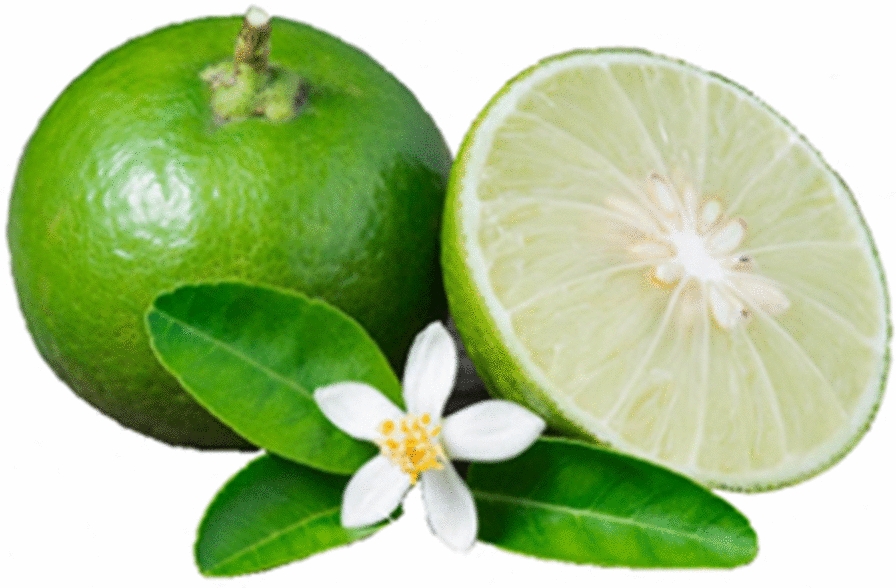
*Citrus aurantiifolia* fruit (Lime), flower and leaf

*Citrus aurantiifolia* (Fig. 1) is a perennial small evergreen tree which can grow to a height of 3–5 m, with dense ramifications and whose surface is covered with rigid thorns. It is classified in the Rutaceae family, genus Citrus. The twigs have a quadrangular shape, and the leaves are elliptical or oval with serrated margins varying in color from yellow-green to dark green. The flowers are yellowish-white, consisting of 4–5 petals [1].

The fruit, commonly called lime, has an ellipsoidal shape, with a large diameter between 3 and 5 cm, initially green in color, but yellow when ripe. The pulp is greenish yellow and produces an acidic but very perfumed juice. These fruits contain few white seeds [1].

Lime is a fruit whose use is increasingly widespread in the world, both as a fresh fruit for consumption and preparation of juices or drinks. It is also used in the preparation for jams and candies. The essential oil obtained from the peel is widely used in the pharmaceutical and cosmetic industry, for drugs, perfumes, soaps, body lotions, but also in the preparation for detergents or to flavor foods or drinks. The peel is also widely used, especially in cooking [2]*.*

This fruit contains coumarins, carotenoids, alkaloids and numerous other constituents, each of which containing numerous pharmacological properties, including anticancer properties [2].

In the literature there are several cases of adverse reactions to lime, most of which deriving from contact with the peel or juice of the fruit and whose manifestation is mainly expressed in the skin, in the form of dermatitis or phytophotodermatitis.

Contact dermatitis is a skin-state alteration characterized, in the acute phase, by the appearance of erythema, itch and vesiculation. These clinical manifestations are induced by exposure of the skin surface to external agents.

The substances that can induce contact dermatitis may be irritant or allergenic [3].

The term “contact dermatitis” includes: Irritant contact dermatitis (ICD), allergic contact dermatitis (ACD), contact urticaria (CU), protein contact dermatitis (PCD), phototoxic contact dermatitis (PTCD), photoallergic contact dermatitis (PACD), systemic contact dermatitis (SCD) [4].

The etiopathogenesis of these types of dermatitis are different. The aim of this study was to collect and review the published studies and cases of adverse reactions to *Citrus aurantiifolia*.

### Food chemistry

In order to understand the pathogenesis of adverse reactions to *C. aurantiifolia*, below we focus on the analysis of the potentially toxic components responsible for the clinical manifestations to lime.

*Citrus aurantiifolia* contains high levels of cumarins and furocoumarins [5]. A gas chromatography–mass spectrometry analysis shows that *Citrus aurantiifolia* oil contains a high number of monoterpenes (83.93%), in particular d-limonene (40.92%) and Citral (27.46%) are the most relevant [6].

### Cumarins and furocoumarins

The main cumarins and furocoumarins contained in the peel and flash of Citrus auratiifolia are 5,7-dimethoxycumarin (Limettin), Psoralen, 5-Methoxypsoralen (Bergapten), 8-Methoxypsoralen (Xanthotoxin) and Isopimpinellin. In addition to Rutaceae, they are common to other plant families such as Umbelliferae, Moraceae, Cruciferae and Ranunculaceae.

**Fig. 2:**
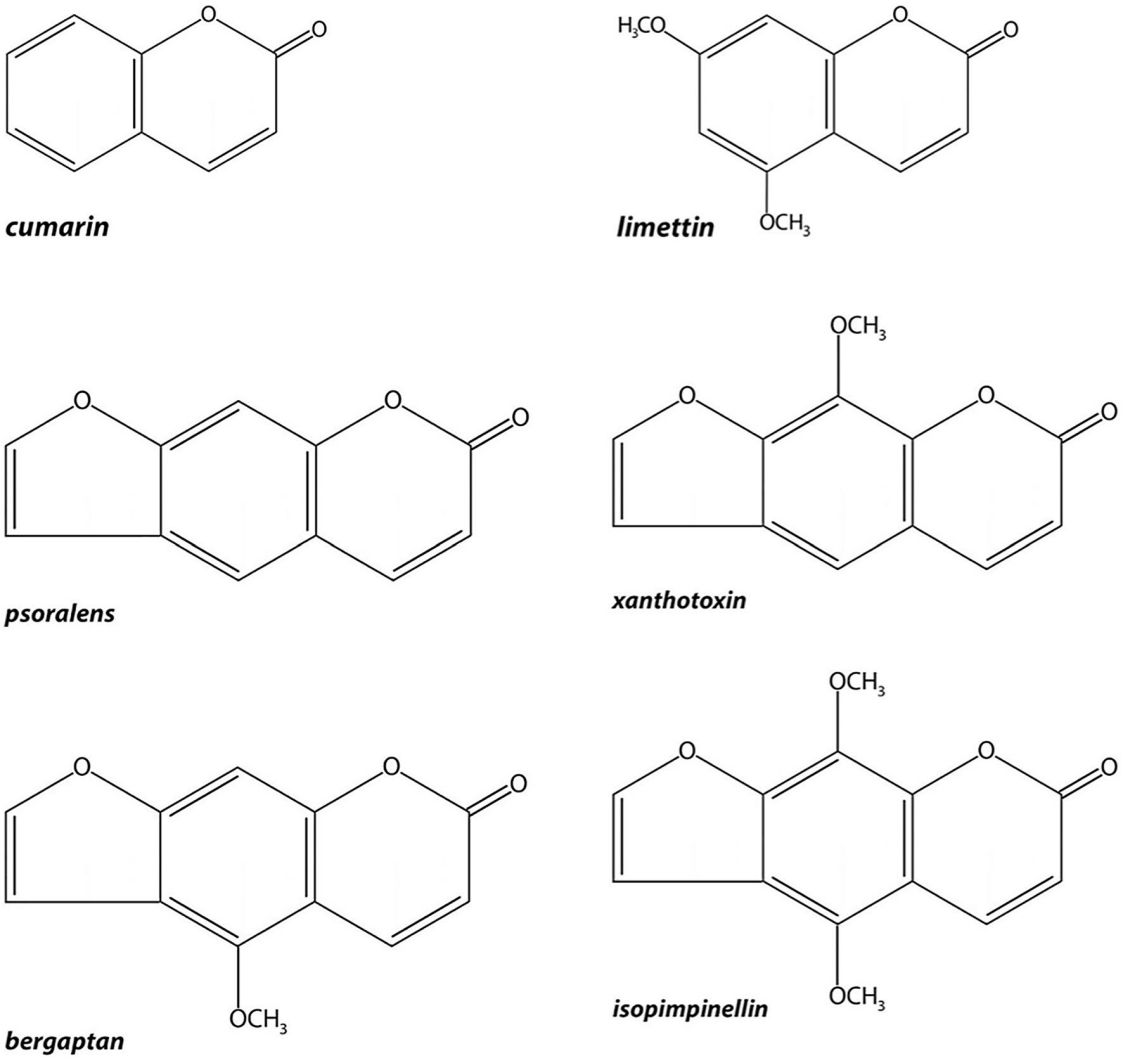
Cumarins and furocumarins chemical structures

Coumarins represent a heterogeneous group of natural heterocycles characterized by high chemical diversity (Fig. 2). All naturally occurring coumarins derive from 5,6-benzo-2 pyrone.

Furocoumarins are natural compounds composed by a basic coumarin structure and a furan ring (Fig. 2); they are usually divided into psoralen-type (linear compound) and angelicin-type (angular compound) [5]*.*

The levels of furocoumarins are higher into peel than into flesh of citrus fruit [5].

The main manifestation due to coumarins and furocoumarins is phytophotodermatitis, a phototoxic reaction.

On the skin surface these compounds are excited into a reactive state after exposure to UV radiation and causes a direct toxic effect [7]. These photo-toxic effects occur independently of the host’s immune system [7] without a prior sensitization [8]. Two types of phototoxic reactions have been described: the first one, oxygen-independent, resulting in an inhibition of DNA synthesis; the second one, oxygen-dependent, resulting in an epidermal, dermal, and endothelial cell membrane damage.

At the same time there is an abnormal melanocytes response to UV rays, with an increase of melanosome production of pigment in the oxygen-independent reaction and a melanocytes injury, with pigments’ release in the oxygen-dependent reaction (7). These reactions are intensified by heat, sweating and wet skin [8].

Not all cumarins and furocoumarins have the same phototoxic action.

Limettin is not phototoxic at 1% on human skin [9]*.*

Psoralens (linear compound) are more phototoxic than angelicin (angular compound) [10], in particular Bergapten (5-MOP), the most potent phototoxin [7], and Xantotoxin (8-MOP) causes acute and severe dermatitis [10]*.*

### D-limonene

**Fig. 3:**
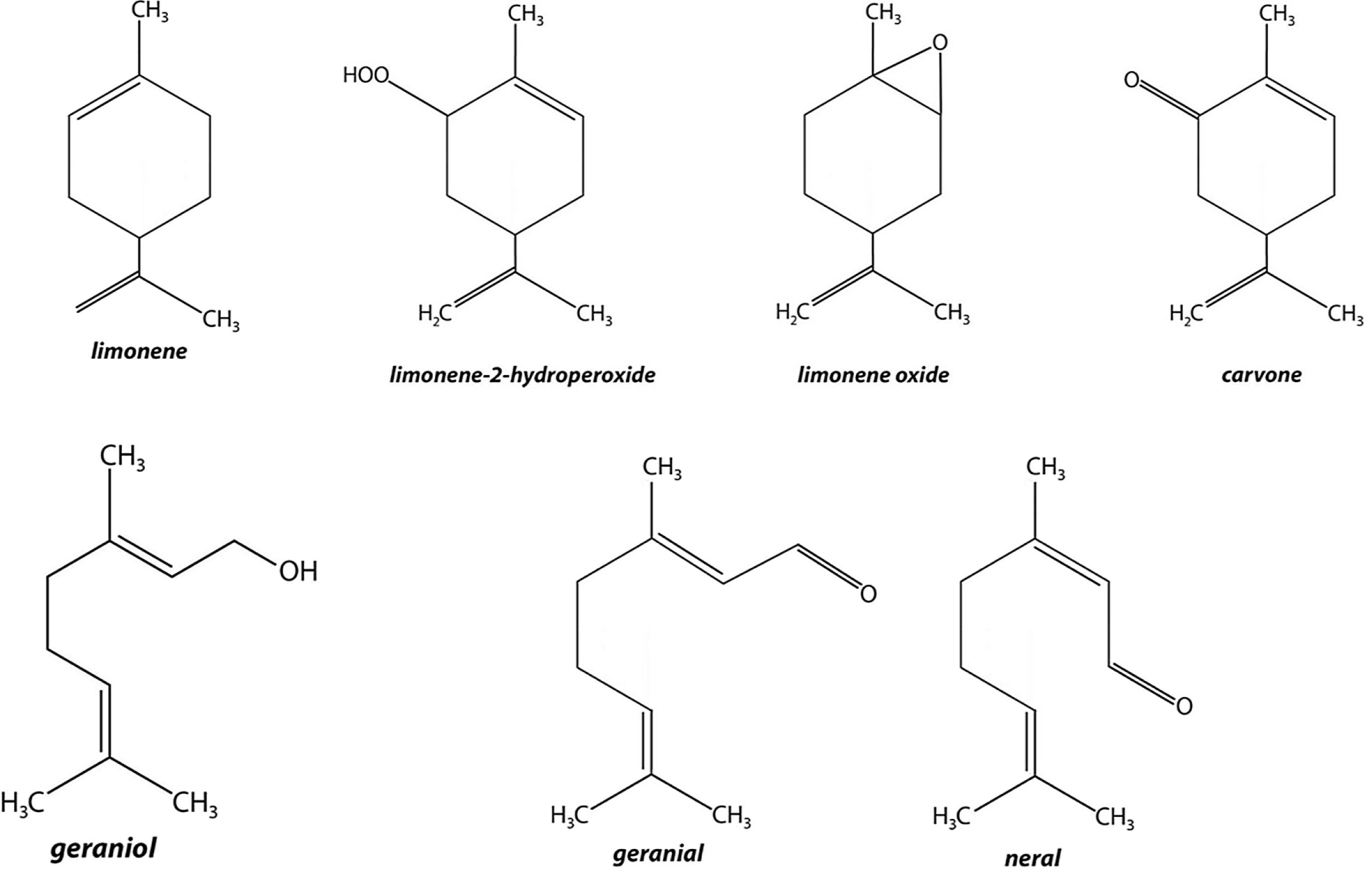
Chemical structure of the main haptens of *C. aurantiifolia*

Limonene is a terpen contained mainly in the fruits of the genus citrus. It can be found in 3 forms: d-Limonene, l-Limonene and the racemic mixture dl-Limonene (Fig. 3) [11].

Peel oil from citrus fruit mainly contains d-limonene, which is regarded as a skin-irritant substance but not an allergenic one in this form. It becomes an allergen after a spontaneously autoxidation process on air exposure, producing Limonene oxide, Limonene Hydroperoxides and R-Carvone (Fig. 3) [12].

De Groot suggested that testing limonene hydroperoxides 0.3% and 0.2% in petrolatum could detect more sensitizations than commonly used fragrances. However, we do not yet know the optimal concentration to use, as there may be some false positives [11].

Limonene also causes respiratory disease, especially in people with bronchial hyperreactivity, because it is a non-specific airway irritant [13].

### Geraniol and citral

Geraniol and citral are monoterpenes (Fig. 3) widely used in the creation of fragrances, cosmetics and hygiene products. In addition to the lime peel oil, geraniol is also found in many other natural oils, such as rose and citronella oil. Citral is the main component of lemongrass oil but is also found in lime leaf and peel oil [14, 15].

The citral is made up of a mixture of two aldehydes, the geranial and the neral (Fig. 3). These often result from the oxidation of geraniol. Hagvall et al. in a 2019 study have highlighted the superiority of patch tests with oxidized geraniol, citral, geranial or neral, compared to the classic fragrances mix currently used [15].

## Methods

We have carried out a Medline search of the case reports about Lime’s adverse reactions. Our analysis included all articles in English language, published until May 2021.

We used the keywords “*Citrus aurantiifolia*”, “*hypersensitivity*”, “*dermatitis, contact*” and “*urticaria*”.

## Results

The Medline search identified 31 articles reporting adverse reactions to lime for a total of 39 cases: 35 of the described reactions are phytophotodermatitis, 3 cases are about contact allergic dermatitis and only 1 case concerns proteins contact dermatitis.

Among 35 cases of phytophotodermatitis (Table 1), 65.71% [23] are young women, with an average age of 23 years old. The male patients [9] have an average age of 20 years old. Of two cases, gender and age are not specified. Age of a man is not specified in a case. In the reported cases we have a sensible difference in the mean age between men and women which became greater not considering the older patient for both. In this last situation the average age in men decreases from 20 to 14.5 years significantly creating a gap of 7 years between the mean age between men and women.

**Table 1:** Characteristics of patients affected by phytophotodermatitis

No of patients	35
Gender	9 M 23 F 2 unknowns
Type of contact	2 ingestions 33 skin contact
Skin lesions	Rash Bullous reaction Vesicles Blisters
Timing	Between 24 h and 120 h after the contact

In most of these cases, the lesions appeared after more than 24 h (36–72 h). In one of the case reports, a clear correlation between lime and phytophotodermatitis cannot be demonstrated, as the patient reported that she ingested the lime and painted her face with pigments derived from an unidentified plant root. Subsequent lesions appeared in the same places of application of the pigment [16].

Regarding the allergic reaction to lime, the (3 cases of allergic contact dermatitis, 1 case of protein contact dermatitis) reported affect female sex, with average age of 46.5 years old (Table 2).

**Table 2:** Characteristics of allergic contact dermatitis cases

Author	Sex	Age	Patch test results (Lime's allergens)	Other sensitization	Occupation	Clinical manifestation after Lime contact
Amy Swerdlin	W	54	Lime peel, geraniol, citral	Yes, but not specified	Bartender cutting and squeezing lemons and limes, washing glasses, and mixing drinks	Itchy, burning, and dry hands affecting her web spaces and fingers. Back of hands and wrists spared. More intense lesions in the right hand (right-handed)
Michelle A. Thomson	W	52	Lime peel, geraniol	Myroxylon pereirae, fragrance santolite resin, geranium oil, rose oil Bulgarian	NA	Eczematous eruption on the lips, left corner of the mouth and left chin. There was also eczema affecting her eye-lids
A C Cardullo	W	52	Lime peel, geraniol, citral	Lemon peel, orange peel, neomycin sulfate, p-tertiary butylphenol, thimerosal, fragrance mix	Bartender cutting and squeezing citrus, washing glasses and table tops. Handling money	Hand dermatitis, more severe on the right hand. Hyperkeratosis and fissuring of the palmar and lateral surface of the thumb and index finger, with paronychia

### Phytophotodermatitis

Phytophotodermatitis are cutaneous manifestations, due to phototoxic reactions induced by the exposure to sunlight after contact with some species of plants. The molecular components responsible of this clinical manifestation are coumarins and furocoumarins, contained in the plant families of Rutaceae (lime, lemon, orange), Umbelliferae (parsley, celery, carrot), Moraceae (fig), Cruciferae (mustard) and Ranunculaceae (buttercup).

The first study dates back to 1993, when Goskowicz et al. described 5 cases of phytophotodermatitis caused by contact with lime in children. They presented blistering eruptions of the photo-exposed areas with streak or finger-print patterns. All of them ate, squeezed or cut limes and the manifestations had risen from a few hours to 24 h after exposure to the sun. Sometimes the rash was painful, and it later turned into hyperpigmented macules, which resolved within some months [17]. Ganesh et al. described the development of the clinical manifestations progressing through three stages: it begins with an erythematous rash with burning sensations (stage 1), followed by painful and tense vesicles (stage 2). Then the vesicles/blisters become flaccid and result in skin hyperpigmentation after about a week (stage 3) [18].

**Fig. 4:**
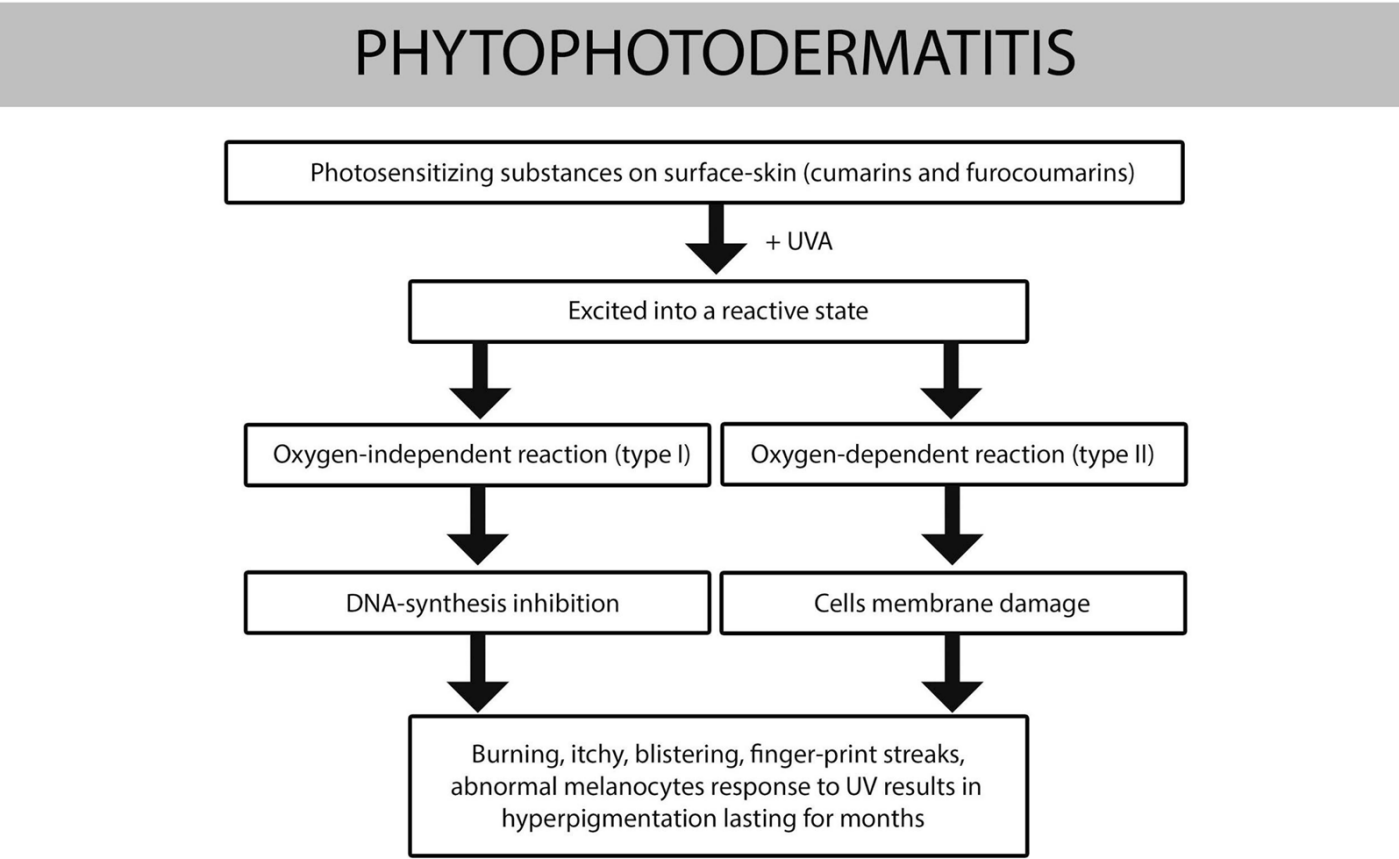
Pathogenesis of phytophotodermatitis

In Fig. 4 are summarized pathogenetic mechanisms. Some case of phytophotodermatitis has been previously misdiagnosed: the manifestations were often confused with Lyme disease, accidental burn injury because of the blisters or child abuse because of the bruise-like and finger-marks pattern [8, 19, 20].

To make differential diagnosis it would be worth executing a punch biopsy of the lesions, which was performed in only one case [21].

### Allergic contact dermatitis

The allergic contact dermatitis (ACD) is an inflammatory reaction mediated by T-cells in patients previously sensitized to an allergen.

Allergens are low molecular weight chemicals, also called haptens. These molecules must be bound to a carrier protein to become immunogenic [22].

Clinical manifestation occurs 24 to 96 h after contact, in the site of contact, with the appearance of a lesion with well-defined edges that can spread locally or at a distance [23].

**Fig. 5:**
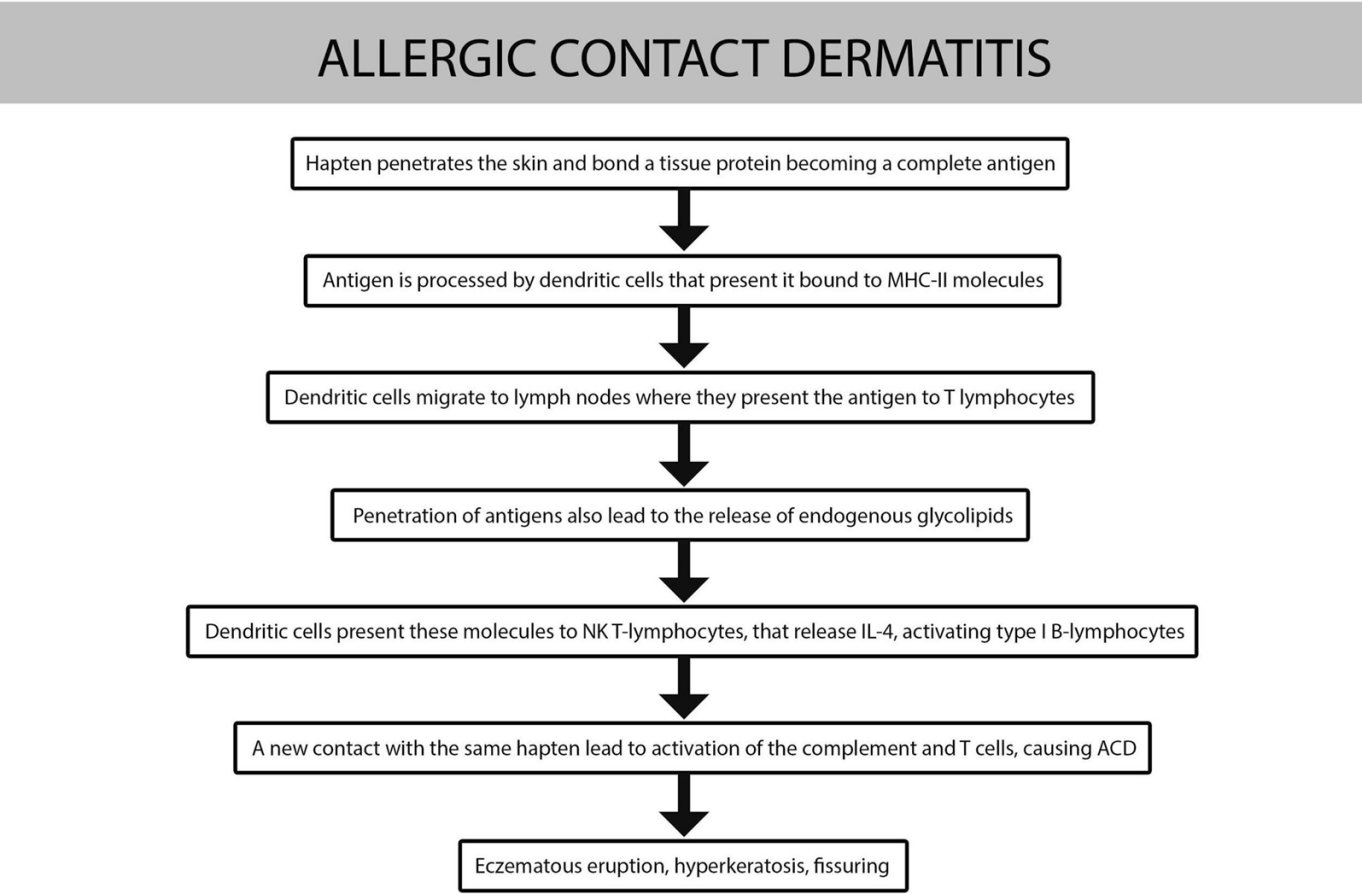
Pathogenesis of ACD

In Fig. 5 pathogenesis is described.

Lime and other Citrus fruit can cause ACD.

D-limonene is the main constituent of peel oil from citrus fruit and experimental studies proved that the handling of the peel oil forms oxidation products like limonene hydroperoxides, that is an allergenic substance [12]. Geraniol and Citral have been identified as minor allergens [24].

#### Case 1

Swerdlin et al. described a case of lime ACD in a 54 years-old female. Her work consisted in cutting, squeezing lemons and limes, washing dishes, and mixing drinks. She described having itchy, burning, and dry hands daily. The clinical examination showed xerotic hyperlinear palms with rough erythematous plaques on her palms and volar fingers and in most of her web spaces. Her dorsal hands and wrists were spared. Her right hand was more involved than her left hand and these manifestations improved during weekend and vacations.

Her history was positive for allergic rhinitis, not for asthma and eczema. The patch test was positive for lime peel exocarp and endocarp but negative for d-limonene [24].

#### Case 2

Thomson et al. reported a case of a 52-years old female, who used sucking lime after drinking her gin tonic. She had eczematous eruption on the lips, left corner of the mouth and left chin in the last 4 months. Her history was positive for mild hand eczema and eyelid dermatitis. The patch test performed was positive for myroxylon pereirae, fragrance mix, santolite resin, geraniol 2%, geranium oil, rose oil Bulgarian and lime peel [25].

#### Case 3

The last case has been reported by Cardullo et al. who described a case of a 54 years-old female, bartender, who cut and squeezed citrus fruit (oranges, lemons, and limes). She had severe hand dermatitis, which improved during vacations. They performed 3 sets of patch tests: she was positive to fragrance mix 16% (+++) in set 1, positive for lemon, lime and orange peel (++), but negative for lime, orange and lemon juices in set 2. In the last set of patch tests she was positive for citral 2% and 5%, but negative for d-limonene 5% [26].

### Protein contact dermatitis

Protein contact dermatitis (PCD) is an allergic skin reaction which occurs due to the contact with some proteins (animals, wheat, fruits, vegetables, spices, wood). It was described for the first time by Hjorth and Roed-Petersen in 1976 [27].

It’s difficult to distinguish it from allergic contact dermatitis [28].

Typically, it presents itself on clinical observation as a form of chronic dermatitis, mainly localized on the backs of the hands and on the fingers and often associated with paronychia [29].

A clinical feature is the immediate appearance of itching, erythema, or blisters, after contact with the responsible protein.

**Fig. 6:**
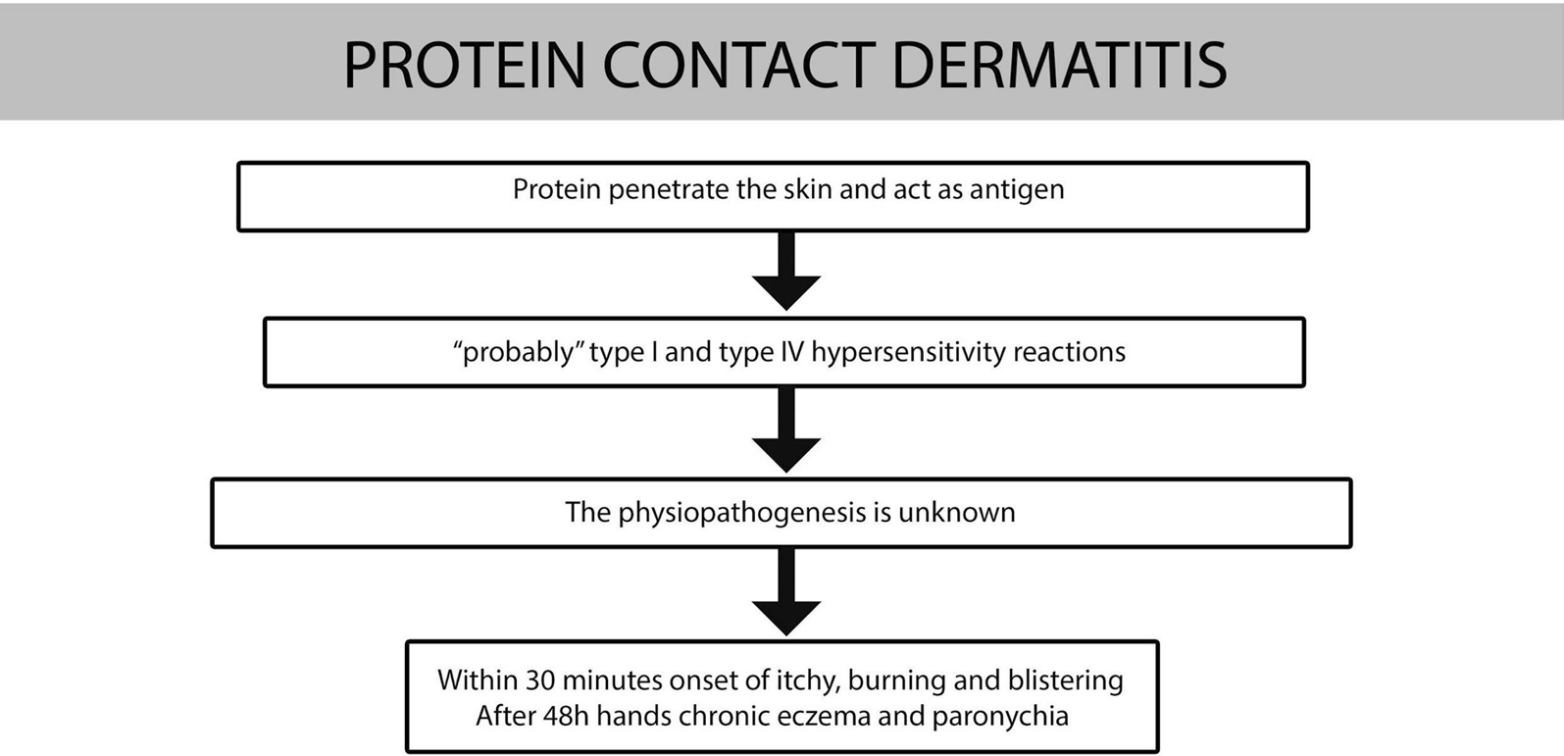
Pathogenesis of protein contact dermatitis

Patch-test is typically negative, conversely, the skin-prick test and the scratch test results are positive. The pathogenetic mechanism is not yet well understood; it could be a combination of a type I and a type IV hypersensitivity reaction (Fig. 6). Alternatively, it could be due to IgE-bearing Langerhans’s cells, in a similar way to what is observed in the pathogenesis of atopic dermatitis [28].

A 28 years-old pastry chef had burning sensation and itch of the hands after contact with lime, pineapple, avocado, drupe, flour and cucumber. Her dermatitis got worse in the last 3 years and spread to face, foreharm, extremities and chest. She referred daily contact with lime zest. Furthermore, she had allergic rhinitis and asthma. Both cutaneous and respiratory symptoms improved during weekends and vacations. She had a positive history of atopic dermatitis, confined to her flexures and well controlled. Prick test was positive for dust mites, cat and horse epithelium, lime, apple, avocado, pineapple, and kiwi. All Patch tests performed gave negative results. RAST (radioallergosorbent test) was positive for latex, hazelnut, almond, lemon, peach, wheat, corn, kiwi fruit, avocado and macadamia nuts [30]*.* The main diagnostic suspicion is protein contact dermatitis but were made other differential diagnoses like contact urticaria caused by latex and fruit or by corn, atopic eczema, and Immediate hypersensitivity to wheat.

## Discussion

Literature analysis revealed only contact reactions to *Citrus aurantiifolia* fruit. There was no evidence of adverse reactions after both ingestion of the fruit or inhalation of tree pollen.

All clinical manifestations were cutaneous with no involvement of other organs.

There are different ways to encounter this fruit, like squeezing, cutting, or picking. In addition to the common use of lime, like in alcoholic and non-alcoholic beverages, cosmetics, and food preparations, it is important to focus on other ways to use this fruit, for example as a natural remedy [19, 31].

Many of the cases described concern patients exposed to *Citrus aurantiifolia* for occupational reasons, such as bartenders, gardeners, or pastry chefs.

Adverse reactions to lime are mainly phytophotodermatitis, few cases are allergic contact dermatitis (ACD) and only one case is described as protein contact dermatitis (PCD). The etiopathogenesis of phytophotodermatitis is well known. Both flesh and peel of *C. aurantiifolia* contain coumarins and furocoumarins, the main responsible of phytophotodermatitis. The features of the lesions make this type of dermatitis easily misdiagnosed. Other differential diagnoses must be considered, such as physical violences or burns. Most reported cases have happened in tropical areas, so the duration of the exposure to UVA and the latitude at which it occurs must be considered as significant environmental factors. Ji Young Choi et al. suggest the importance of the energy level of the UVA ray: they have made up a skin provocation test applying Finn chambers full of lime extract on the volunteers’ back and after two hours they have irradiated with various doses of UVA; after 3 days this test has showed the erythema appeared only on skin exposed to an energy level as low as 10 J/cm^2^ [21].

Therefore, to make a correct diagnosis, a careful medical history cannot be ignored: employment status, history of recent trips to tropical areas and consumption of drinks containing lime are fundamental to collect. Age and sex factors should be taken into consideration, as phytophotodermatitis appear to be more common in young women.

In men it seems to be less frequent and occurs mainly in the pediatric age. However, data related to the cases of phytophotodermatitis in males are limited (Fig. 2).

Regarding allergic reactions to lime, type IV hypersensitivity is the main pathogenetic mechanism (ACD). However, lime could be responsible for protein contact dermatitis (PCD), which has a different pathogenetic mechanism than ACD, not yet well known. To discriminate between the two forms is needful to know the timing of onset of the lesions. These usually appear 24–96 h after exposure, in the contact point and with possible remote localization, in ACD. Late onset of lesions and the pathogenetic mechanisms justifies patch test as the main diagnostic test in ACD, that is typically positive. In PCD there is an acute phase with erythema, itching and blisters appearing immediately in the contact point; secondly, we can find signs of eczematous chronic dermatitis. The negativity to the patch test with a positivity to the skin prick test is a feature of PCD, which suggests that the pathogenetic mechanism seems to be a combination of immediate type I and delayed type IV hypersensitivity.

D-limonene is recognized as the major allergen of the genus Citrus but in the 3 cases reported in our analysis patch tests were negative to D-limonene and positive to Geraniol and Citral, that are minor allergens [24].

According to literature there is evidence regarding the greater sensitivity of the patch test performed with Citral or its singular components (Geranial and Neral) and the oxidized forms of Limonene and Geraniol compared to baseline fragrance materials currently used (11, 15).

Regarding PCD, the only case reported is controversial. The onset of clinical manifestation and the diagnostic tests argue in favor of this diagnosis, but the patient had multiple sensitizations and she was exposed to different allergens at the same time; so it’s difficult to identify the real culprit of the allergic reaction. In the end, chronic exposure to multiple allergens in an occupational environment confuses the clinical scene.

## Conclusions

Increasingly widespread use of lime in the world (as a fresh fruit, in alcoholic and non-alcoholic beverages, in the pharmaceutical and cosmetic industry), attention must be paid to the potential increase in adverse reactions to lime in the coming years.

In some people and in specific environmental conditions lime can cause skin reactions such as phytophotodermatitis, allergic contact dermatitis (ACD) and protein contact dermatitis (PCD).

A thorough history and examination can help identify lime as a cause of these clinical skin manifestations enabling implementation of successful avoidance strategies. Anamnestic collection must include questions about occupational exposure, contact with lime during recent trips in tropical areas, timelapse between contact with lime and onset of clinical manifestations, different and unusual ways to encounter this fruit.

After all, it could be useful to evaluate the use of alternative preparations to those commonly used in patch tests. To increase the sensitivity of this diagnostic test, it would be more appropriate to use individually the molecular components (oxidized D-limonene, oxidized geraniol, citral, neral and geranial).

## Data Availability

Not applicable.
